# Systematic Review of Otologic and Neurotologic Surgery Using the 3-dimensional Exoscope

**DOI:** 10.1097/ONO.0000000000000024

**Published:** 2022-12-02

**Authors:** Harry Chiang, Leila Ledbetter, David M. Kaylie

**Affiliations:** 1 Department of Head and Neck Surgery and Communication Sciences, Duke University Medical Center, Durham, North Carolina; 2 Medical Center Library and Archives, Duke University School of Medicine, Durham, North Carolina.

**Keywords:** Exoscope, Ergonomics, Neurotology, Otology, Skull Base Surgery, Technology

## Abstract

**Objective:**

The 3D exoscope is an emerging technology that has been met with success in neurosurgery and is now increasingly used in otologic and neurotologic surgery. There is currently no consensus on its safety, efficiency, and utility, compared to the traditional microscope for these procedures. This systematic review aims to evaluate the use of the 3-dimensional (3D) exoscope for otologic and neurotologic surgery.

**Databases Reviewed:**

MEDLINE/PubMed, Web of Science, Scopus, and EMBASE.

**Methods:**

A systematic search of the databases was conducted for otologic and neurotologic surgery using the 3D exoscope. English language papers with no limit on the date of publication were considered. Inclusion criteria: full articles studying otologic or neurotologic/skull base surgery using exoscopes. Exclusion criteria: non-otologic surgery and non-neurotologic/skull base surgery, exclusive use of the traditional microscope, editorials, video reports, and letters. Two authors independently reviewed papers for inclusion; discrepancies were settled by consensus. Extracted variables included: number of patients, types of surgical procedures, operative and postoperative complications, setup and operative time, and visualization and ergonomic rating.

**Results:**

Six articles containing 128 surgical cases (103 exoscopic and 25 microscopic) were analyzed. Of the exoscopic cases, 21% were surgeries for chronic ear disease, 5% were cochlear implants, and 74% were lateral skull base procedures encompassing a wide variety of approaches.

**Conclusion:**

Based on preliminary studies, the exoscope appears to be comparable in safety, visualization, and efficiency compared to the operating microscope, with the potential for increased comfort and ease of use.

The binocular microscope has remained the gold standard for otologic and neurotologic surgery for decades, providing clarity, illumination, and high magnification. However, poor ergonomics, limited focal range, and bulky size are frequent complaints (1). In recent years, a new generation of extracorporeal microscopes, or “exoscopes,” have entered clinical practice. The exoscope consists of a high-definition rigid rod-lens telescope suspended above the surgical field (2). Three-dimensional (3D) video is projected in real-time onto a monitor, allowing the surgeon to operate with a heads-up posture while wearing 3D glasses (3). A large depth of field reduces the need for refocusing. Shared 3D images on the monitor and compact size of the exoscope are thought to improve teaching, communication, and instrument exchange. Initially met with success in neurosurgery, the exoscope is now increasingly used in otology and neurotology as a complement or replacement for the microscope. These are not insubstantial investments for hospitals to make, and the purchasers may be weary of a new technology that may or may not be appropriate for their surgeons. A systematic review of the literature may provide information for surgeons to make an informed decision on whether to utilize this new technology. In this systematic review, we assess the safety, efficiency, and utility of 3D exoscopes in otologic and neurotologic surgery, and explore its advantages and limitations compared to the binocular microscope.

## METHODS

This systematic review was written according to the 2020 Primary Reporting Items for Systematic Reviews and Meta-analyses (PRISMA) Guidelines (4).

### Databases and Search Strategy

A systematic search of the MEDLINE/PubMed, Web of Science, Scopus, and EMBASE databases was conducted for otologic and neurotologic surgery using the 3D exoscope with no limit on the date of publication. The search was developed and conducted by a professional medical librarian to include English language studies with keywords and subject headings representing exoscope technology and otologic or neurotologic surgery. The methodology was obtained from the Cochrane Handbook for Systematic Reviews of Interventions (5). Search terms included: “Otologic Surgical Procedures,” “Skull Base,” “Neurotology,” “Otologic, Otology,” “Skull base,” “Cranial Base,” “Basis Cranii,” “Base of Skull,” “Basicranium,” “Cranial Fossa,” “Infratemporal Fossa,” “Neurotology,” “Otoneurology,” “Neuro-Otology,” and “Neuro Otology,” which were cross-referenced against “Exoscope,” “Exoscopes,” “Exoscopic,” “Video Assisted Telescope Operating Monitor,” “VITOM,” and “Video Telescope Operating Monitor.”

All identified studies were uploaded into Covidence, a software system for managing systematic reviews. After duplicates were removed by the software, 2 authors independently screened the articles and excluded those that were not relevant or appropriate for this review. Discrepancies were settled by consensus. Primary eligibility criteria were English language papers with no limit on the date of publication regarding the use of exoscopes with otologic or neurotologic surgery. Studies involving non-otologic and non-neurotologic/skull base surgery or exclusive use of the traditional microscope were excluded. Editorials, video reports, letters, and articles without available full text were also excluded.

### Risk of Bias Assessment

The Newcastle-Ottawa Quality Assessment Form for Cohort Studies was used to assess the risk of bias for each of the articles.

### Data Extraction

After final screening, the articles were divided into 2 groups: those using the exoscope exclusively and those using both the exoscope and the microscope. Extracted variables included: number of patients, types of surgical procedures, operative and postoperative complications, setup and operative time, visualization and ergonomic rating, and exoscope system used. Surgical procedures were grouped into 1 of 3 categories: those for chronic ear disease, cochlear implantation, and lateral skull base surgery. Bilateral surgeries were considered as separate procedures. Data were analyzed qualitatively.

## RESULTS

### Study Selection

**FIG. 1.**
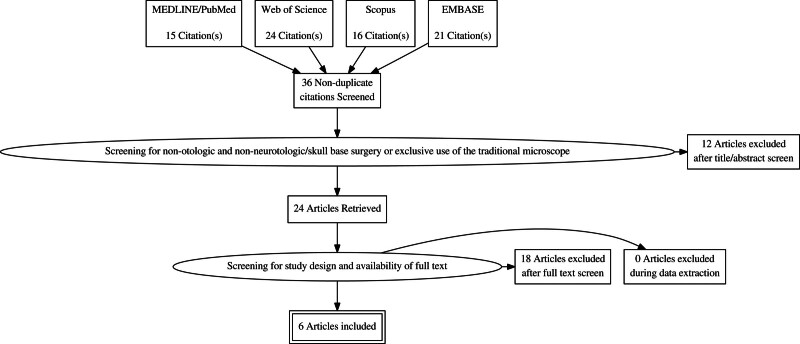
PRISMA flowsheet for article selection criteria. Seventy-six articles were identified and narrowed down to 6 final articles included in this study. PRISMA indicates Primary Reporting Items for Systematic Reviews and Meta-analyses.

Seventy-six articles were identified with the search and forty duplicates were removed by the software. The remaining 36 citations were then screened independently by 2 authors. Twelve articles were excluded due to not being relevant to the purpose of the review (10 articles not related to the clinical use of the exoscope and 2 articles not using the exoscope in the context of otologic or neurotologic surgery). Additionally, 10 articles were excluded for incorrect study design, 4 for unavailable full text, 1 for focusing on the wrong patient/intervention population, 2 for being video reports, and 1 for being a missed duplicate. After all screening, a total of 6 articles were included in this review. Two of these articles were deemed to be fair quality, and 4 were deemed to be poor quality on the Newcastle-Ottawa Quality Assessment Form. All 6 articles were of level 3 evidence, based on the Centre for Evidence Based Medicine guidelines (6). The study selection is presented by flowchart as per PRISMA guidelines (Fig. 1).

### Surgical Procedures

Of the 6 total articles, 2 reported cases using only the exoscope and 4 reported cases using both the exoscope and microscope. The articles covered a time frame of 2019 to 2021 and included a total of 128 surgeries with subjects ranging between 15 and 81 years of age. Eighty percent of the total 128 cases were exoscopic (n = 103) and 20% were microscopic (n = 25). Of the exoscopic cases, 21% were surgeries for chronic ear disease (n = 22), 5% were cochlear implants (n = 5), and 74% were lateral skull base procedures (n = 76). Of the microscopic cases, 44% were for chronic ear disease (n = 11), 8% were for cochlear implants (n = 2), and 48% were for lateral skull base procedures (n = 12). The chronic ear disease procedures described in these articles included myringoplasty, tympanoplasty, mastoidectomy, and tympanoplasty with mastoidectomy (3,7–9). Lateral skull base surgeries in these articles included many skull base approaches: infratemporal (1 microscopic, 1 exoscopic), transcanal (1 microscopic, 3 exoscopic), transcochlear (1 microscopic, 3 exoscopic), translabyrinthine (1 microscopic, 1 exoscopic), retrolabyrinthine (2 microscopic, 2 exoscopic), middle cranial fossa (1 microscopic, 1 exoscopic), subtotal petrosectomy (5 microscopic, 6 exoscopic), lateral temporal bone resection (10 exoscopic), subtotal temporal bone resection (45 exoscopic), and suboccipital craniotomy (2 exoscopic) (1,8–10). Pathologies included cholesteatoma, vestibular schwannoma, paraganglioma, chondrosarcoma, malignancies of cutaneous or salivary origin, Meniere’s disease, facial paralysis, and temporal lobe encephalocele (1,8–10). Eighty-three of the exoscopic cases used the Karl Storz VITOM 3D (KARL STORZ, Tuttlingen, Germany), 6 used the Synaptive Brightmatter (BrightMatter Servo, Synaptive Medical, Ontario, Canada), and 16 used the Olympus ORBEYE (OLYMPUS, Tokyo, Japan). Four of the 6 included studies described the operating room setup with the exoscope—in all of these cases, a heads-up display was used at approximately eye level. The details of each study are summarized in Table 1.

**TABLE 1.** Study populations, procedures, and metrics

Study	EX: *N*	OM: *N*	Age	Exoscope	Procedure distribution	Relevant complications	Visualization	Ergonomics	Setup/OR time
Garneau (2019)	6	N/A	20–69	Brightmatter	6 lateral skull base (4 vestibular schwannoma resection, 2 temporal lobe encephalocele repair)	1 postoperative facial paresis, 1 CSF leak immediate postop subsequently repaired	No	Yes	Yes
Smith (2019)	11	N/A	27–81	VITOM 3D, ORBEYE	6 chronic ear diseases (4 mastoidectomies, 2 transcanal approaches); 2 cochlear implants; 3 lateral skull base (2 suboccipital craniotomies, 1 subtotal petrosectomy)	None	Yes	Yes	No
Kanzaki (2020)	6	N/A	30–78	ORBEYE	5 chronic ear diseases (5 tympanoplasties); 1 cochlear implant	N/A	Yes	No	Yes
Raymond (2020)	55	N/A	Mean 67	VITOM 3D	55 lateral skull base (45 subtotal temporal bone resections, 10 lateral temporal bone resections)	13 microscopic positive margins (24%): authors remarked comparable to that with the operating microscope in the literature)	Yes	Yes	Yes
Rubini (2020)	12	12	11–79	VITOM 3D	EX: 12 lateral skull base (1 infratemporal, 1 transcanal, 1 translabyrinthine, 5 subtotal petrosectomy, 2 retrolabyrinthine, 1 middle cranial fossa, 1 transcochlear) OM: 12 lateral skull base (1 infratemporal, 1 transcanal, 1 translabyrinthine, 5 subtotal petrosectomy, 2 retrolabyrinthine, 1 middle cranial fossa, 1 transcochlear)	EX: 3 postoperative facial nerve weakness OM: 1 external canal cul de sac dehiscence, 2 postoperative facial nerve weakness	Yes	Yes	Yes
Colombo (2021)	13	13	15–80 years	VITOM 3D	EX: 11 chronic ear disease (9 tympanomastoidectomy, 1 mastoidectomy, 1 revision myringoplasty); 2 cochlear implant OM: 11 chronic ear disease (9 tympanomastoidectomy, 1 mastoidectomy, 1 revision myringoplasty); 2 cochlear implant	EX: 1 CSF leak OM: None	No	No	Yes

EX indicates exoscope; CSF, cerebrospinal fluid; OR, operating microscope.

### Safety and Complications

Five of the 6 articles logged intraoperative and postoperative complications. Of the 99 exoscopic cases from these 5 articles, only 1 intraoperative complication was reported––a cerebrospinal fluid (CSF) leak which was treated intraoperatively (3). There were no intraoperative complications reported from the 27 microscopic cases (3,9). Of the 99 exoscopic cases, there were 7 postoperative complications and of the 27 microscopic cases, there were 3 postoperative complications. Complications thought to result from surgery included CSF leak (2 exoscopic), facial paresis or worsened postoperative facial nerve exam (4 exoscopic, 2 microscopic), external auditory canal cul de sac dehiscence (1 microscopic), and positive postoperative margins (13 exoscopic) requiring further tumor board discussion and potential adjuvant treatment including radiotherapy (1,3,8–10). The proportion of cases with positive margins was found to be comparable with similar cases when using the operating microscope (10). There was 1 case of vocal cord paralysis and 1 case of ischemic hypoxic brain injury, neither of which were thought to be directly associated with the otologic and neurotologic portions of the surgeries (9,10).

Five studies commented on whether the operating microscope was used for cases assigned to the exoscope group (1,3,8–10). Notably, 4 out of the 99 exoscopic cases from these studies required brief use of the operating microscope, but Rubini et al commented that these cases were found to be during the surgeons’ first encounters with the exoscope (9). A survey also showed that surgeons who had performed 3 or more exoscopic procedures favored the exoscope over the microscope compared to those who had performed 2 or fewer exoscope procedures (exoscope score 4.53/5 versus 3.58/5, *P* < 0.001). Prior training in both endoscopic and microscopic surgery was found to be helpful for lessening the learning curve for using the exoscope (7). Both exoscope and microscope cases included use of the endoscope on rare occasion for a final check after resection of tumor.

### Setup and Operative Time

Four articles compared the efficiency of setup and operative time between exoscopic and microscopic surgery. One study reported no difference in setup time from 1) time at entrance to the operating room until incision, and 2) time from end of induction to incision (*P* = 0.90, *P* = 0.76) (3). In a survey, setup of the exoscope was found to be slightly more involved than that of the microscope (3.29/5) with 3 indicating comparable difficulty of setup (7). In 2 studies that matched procedures by type of surgery, there was no difference in duration of operation or total surgical duration (9,10).

### Visualization, Ergonomics, and Teaching

Visualization of anatomical structures was evaluated in 4 articles. The exoscope was overall found to provide equal if not improved image quality when compared to the microscope (7–10). In 1 study, surgeons agreed that the image quality of the exoscope was better than that of the microscope with an average agreement score of 4.13/5 (with 3 indicating neutral opinion or equivalent image quality between the 2 devices) (7). All 4 articles described excellent visualization and realism of the exoscope images. In 1 study, there was no significant difference in image quality compared to with the microscope (8).

Ergonomics of the exoscope versus the microscope were described in 4 articles. The authors described notable subjective improvement in neck positioning, horizontal gaze, and decreased discomfort or headache when wearing glasses. One article found that neck strain was decreased with the exoscope (*P* < 0.05) when compared with the microscope, but no overall difference in ease of manipulation of overall ergonomics (8).

The exoscope was also subjectively found to be superior for teaching as all members of the operative team share the same 3D high-definition image source (1,7,9,10). In 1 survey, the exoscope was found to be superior to the microscope for teaching with a rating of 4.75/5 with 3 indicating equal teaching (7). Medical students, surgical residents, and fellows anecdotally reported an increased understanding of the anatomy and surgery (10). The exoscope was also described to improve mobility within the operating room due to decreased space occupation compared to the microscope.

## DISCUSSION

Garneau et al. were among the first to describe the use of the exoscope for lateral skull base surgery in 2019, reporting 6 cases of vestibular schwannoma resections and temporal lobe encephalocele repairs via transmastoid transtemporal approach. Since then, studies have expanded on the variety of surgical procedures using the exoscope, from the treatment of chronic ear disease to cochlear implantation and lateral skull base surgery. As such, although the exoscope is a relatively new technology, the surgeries described in this review include a wide breadth of the surgeries performed in the field of otology and neurotology.

In the studies that examined safety and visualization with the exoscope, the image quality was found to be similar to that with the operating microscope. Intraoperative complications were also rare with the exoscope. Although there were more total complications with exoscopic cases than with microscopic cases, based on the far greater number of exoscopic cases included in these articles, the rate of postoperative complications appears to be comparable with that of the microscope. Rarely did exoscopic surgeries require brief use of the operating microscope. In addition, when this did occur, the cases were found to be during surgeons’ first experiences using the exoscope. There appears to be an initial learning curve with the exoscope, but increased exposure may be associated with increased comfort and efficiency using the new technology. Visualization of anatomical structures with the exoscope was found to be at its best for wide surgical apertures such as mastoidectomies, with improved recognition and differentiation of complex structures (8). Some authors commented on possible low lighting in narrow corridors, although new software settings that allow for changes in depth of field when working through speculums, enhance the lighting and clarity of images (8,9). Studies comparing exoscopic and microscopic safety in neurosurgery have also commented on the theoretical benefit of LED lighting in exoscopes producing less heat and thus decreasing the risk of thermal injury compared to the halogen bulbs in microscopes (11).

The primary advantages of the exoscope lie in its overall ease of use compared to the microscope. The exoscope improves mobility within the operating room due to decreased space compared to the microscope. These advantages are likely compounded by improved operative efficiency due to a shared 3D high-definition image source between all team members, which would allow the improved ability for technicians to anticipate instruments for handoff to surgeons. Moreover, due to the shared image source, the exoscope is particularly suitable for use in academic centers with improved teaching for medical trainees ranging from medical students to surgical residents and fellows. Despite being a relatively new technology, the setup and operative time during exoscopic surgery were found to be equivalent to that of microscopic surgery. As a relatively new technology, efficiency with the exoscope is likely to improve further with increased use, whereas the microscope has been a longstanding technology in the field.

Ergonomics for otolaryngologists—and otologists and neurotologists in particular—have increasingly been a concern. Over 70% of otolaryngologists report some level of neck or back pain (12). For otologists and neurotologists, this is attributed to neck flexion due to prolonged operative microscope use (13). Although the ergonomics of specially engineered chairs have been explored, chronic neck flexion associated with microscope use has not been addressed (14). Although adjustable positioning of the microscope can improve overall ergonomics during use, the advantage of the exoscope lies in its ability for free neck movement without position-dependent optics and the ability to maintain horizontal gaze during surgery. Occupational hazards are likely to decrease with use of the exoscope, although long-term studies will provide further insight into the advantages of exoscopic surgery on the long-term musculoskeletal health of otologists and neurotologists.

When discussing complementary or alternative surgical technology, a discussion of cost is paramount. Although the authors’ institution does not own an exoscope, studies have demonstrated that the cost of commercially available 3D exoscopic systems, though encompassing a wide range, is generally comparable to that of microscopes (11). Exoscopic systems also have the benefit of sterilization and thus avoid the operating cost of disposable drapes associated with microscopes.

The primary advantages of the exoscope lie in its overall ease of use with regards to efficiency, teaching, and ergonomics, compounded by excellent visualization and comparable safety to the microscope.

## LIMITATIONS

There are several limitations to this review. Few studies, particularly those with objective or quantifiable data, are available for review as the exoscope is a new technology in the field of otology and neurotology. First, larger-scale studies investigating the use of the exoscope and operating microscope, matched by surgical technique and pathology, will be necessary to generate a sufficiently powered comparison between the 2 technologies. Moreover, there is little overlap in the metrics used in each of the articles. As interest in exoscopic surgery grows, questions regarding exoscopic and microscopic surgery will likely become more focused and refined, creating an increased overlap in metrics with potential for meta-analysis. The literature is currently unable to support this. For example, using standardized methods for evaluating ergonomics, such as the Rapid Entire Body Assessment or Rapid Upper Limb Assessment surveys could help to elucidate the differences between exoscopic and microscopic surgery (15,16). The articles described in this review are also at risk of publication bias. Because there is limited data regarding exoscopic surgery in otology and neurotology, findings that reject the null hypothesis are more likely to gain traction in the literature. Exoscope technology is also evolving—a combined microscope and exoscope platform is also possible, but this technology was not used in the included articles. This review thus focuses exclusively on the exoscope in comparison to the microscope. Despite these limitations, this review shares the collective experience of 3D exoscopes in otologic and neurotologic surgery and provides a starting point for understanding its advantages and disadvantages. Future studies will benefit from refining their questions regarding exoscopic in otology and neurotology and standardizing their methods.

## CONCLUSIONS

Based on preliminary studies, the exoscope appears to be comparable in safety, visualization, and efficiency compared to the operating microscope, with the potential for increased comfort and ease of use. As such, the exoscope may play an important role in the future of otology and neurotology, although further investigation is necessary to clarify its role.

## ACKNOWLEDGMENTS

The authors gratefully thank the Duke University Medical Center Library for assistance in developing and conducting the online database search for this systematic review.

## FUNDING SOURCES

None declared.

## CONFLICT OF INTEREST

None declared.

## DATA AVAILABILITY

The datasets generated during and/or analyzed during the current study are publicly available.
